# Immobilization of Horseradish Peroxidase onto Montmorillonite/Glucosamine–Chitosan Composite for Electrochemical Biosensing of Polyphenols

**DOI:** 10.3390/bios14060278

**Published:** 2024-05-29

**Authors:** María Belén Piccoli, Florencia Alejandra Gulotta, Mariana Angélica Montenegro, Noelia Luciana Vanden Braber, Verónica Irene Paz Zanini, Nancy Fabiana Ferreyra

**Affiliations:** 1 Instituto de Investigaciones en Fisicoquímica de Córdoba (INFIQC-UNC-CONICET), Departamento de Fisicoquímica, Facultad de Ciencias Químicas, Universidad Nacional de Córdoba, Córdoba X5000HUA, Argentina; belen.piccoli@unc.edu.ar; 2 Independent Researcher, Santiago del Estero G4206XCP, Argentina; florgulotta@gmail.com; 3 Centro de Investigaciones y Transferencia de Villa María (CIT-VM), Consejo Nacional de Investigaciones Científicas y Técnicas (CONICET), Universidad Nacional de Villa María (UNVM), Villa María X5220XAO, Argentina; mamontenegro@conicet.gov.ar (M.A.M.); noeliavanden@gmail.com (N.L.V.B.); 4 Instituto de Bionanotecnología del NOA (INBIONATEC), Consejo Nacional de Investigaciones Científicas y Técnicas CONICET, Universidad Nacional de Santiago del Estero (UNSE), Santiago del Estero G4206XCP, Argentina

**Keywords:** water soluble chitosan derivatives, glucosamine–chitosan, sodium montmorillonite, horseradish peroxidase, polyphenols

## Abstract

Glucosamine–chitosan synthesized by the Maillard reaction was combined with montmorillonite to obtain a nanohybrid composite to immobilize horseradish peroxidase. The material combines the advantageous properties of clay with those of the chitosan derivative; has improved water solubility and reduced molecular weight and viscosity; involves an eco-friendly synthesis; and exhibits ion exchange capacity, good adhesiveness, and a large specific surface area for enzyme adsorption. The physicochemical characteristics of the composite were analyzed by infrared spectroscopy and X-ray diffraction to determine clay–polycation interactions. The electrochemical response of the different polyphenols to glassy carbon electrodes modified with the composite was evaluated by cyclic voltammetry. The sensitivity and detection limit values obtained with the biosensor toward hydroquinone, chlorogenic acid, catechol, and resorcinol are (1.6 ± 0.2) × 10^2^ µA mM^−1^ and (74 ± 8) nM; (1.2 ± 0.1) × 10^2^ µA mM^−1^ and (26 ± 3) nM; (16 ± 2) µA mM^−1^ and (0.74 ± 0.09) μM; and (3.7± 0.3) µA mM^−1^ and (3.3 ± 0.2) μM, respectively. The biosensor was applied to quantify polyphenols in pennyroyal and lemon verbena extracts.

## 1. Introduction

Natural polyphenols constitute a group of compounds of great interest, mainly because their consumption is associated with a lower risk of suffering from cancer and/or cardiovascular or degenerative diseases. These compounds have important antioxidant, anti-inflammatory, antitumor, and antimutagenic properties [1]. In this context, their quantification in plant extracts is crucial both in determining the beneficial potential of these extracts in human health and in the food industry for the development of products with high polyphenolic compound content. Several methods have been reported for the quantification of polyphenols, among them, UV–visible spectroscopy [2], high-resolution chromatography [3], and mass spectroscopy [4]. However, these methodologies require laborious preliminary stages for sample conditioning and expensive equipment and reagents. In this regard, electrochemical biosensors represent an excellent alternative with advantages such as rapid response, specificity, minimal sample preparation, and less expensive equipment [5].

Chitosan (CHIT) is one of the most used biopolymers in studies and research aimed at the development of electrochemical biosensors due to its versatility and unique properties. Furthermore, numerous research efforts are continually underway to explore new CHIT properties and/or improve existing ones [6,7]. The solubility of CHIT plays a crucial role in its potential applications. In this context, the Maillard reaction (MR) emerges as a safe synthesis methodology that can obtain CHIT derivatives with improved water solubility [8,9]. Despite their improved properties [10], their use in the design and development of electrochemical biosensing platforms is a recent area of interest aimed at capitalizing on their notable characteristics [11]. Due to their hydrophilic nature, CHIT derivatives obtained by MR can be used as dispersing or surface-functionalization agents of nanomaterials to obtain nanohybrid composites with synergic and improved properties.

Within inorganic nanomaterials, clay minerals have been widely used as immobilization matrices for biomolecules due to their mechanical and thermal stability and their ion exchange capacity associated with their chemical structure [12]. It has been proven that clay minerals provide stability to biomolecules against changes in pH and temperature [13]. Montmorillonite (Mt) has been used for the immobilization of proteins such as hemoglobin, polyphenol oxidase, glucose oxidase [14], laccase [15], and horseradish peroxidase (HRP) [16], among others [17,18], proving that Mt constitutes a favorable matrix for the immobilization of enzymes, allowing them to preserve their native structure and, therefore, their bioactivity.

One way to immobilize enzymes using Mt is through ion exchange, replacing its Na^+^ or Ca^2+^ ions with positively charged enzyme molecules. This mechanism is restricted to small-diameter enzymes such as lysozyme, β-glucuronidase, pepsin, hemoglobin, and lactoglobulin [19]. Immobilizing larger proteins requires the use of other strategies. An alternative is the preparation of the nanocomposites of clays and biopolymers such as CHIT, cellulose and its derivatives, starch, alginate, or pectin, among others. Nanocomposites of Mt–biopolymers allow for the formation of films suitable for functionalizing transducers, for example, electrodes, and provide free functional groups for biomolecule binding, allowing long-term stable structures to be obtained [20]. The addition of polycations increases the mechanical stability of clay films and could improve the electrical conductivity at the interface. All of these characteristics are necessary for enzyme immobilization for biosensing purposes [21].

In this work, we combined a CHIT derivative functionalized by MR with glucosamine (GA-CHIT) and sodium montmorillonite (Na^+^-Mt), to obtain a composite with the aim of immobilizing the HRP enzyme and developing an electrochemical biosensor for polyphenol quantification. HRP is a redox enzyme with an isoelectric point between 8.7 and 9.0 [22] that catalyzes the reduction of H_2_O_2_. Polyphenolic compounds can act as electron donors for HRP in the catalytic reduction of H_2_O_2_, and their concentration can be detected from the reduction current of the oxidized products generated by the enzymatic reaction cycle.

**Scheme 1:**
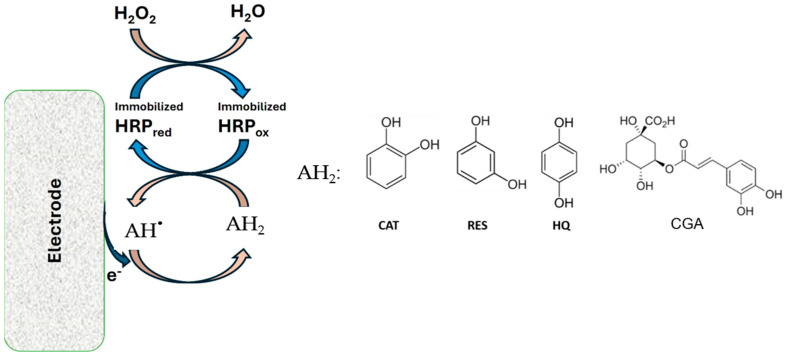
Enzymatic and electrode reaction scheme involved in the response to polyphenols of HRP electrochemical biosensors.

A wide variety of hydrogen-donating compounds—including phenols, indoles, and amines—and sulfonates can be oxidized in the presence of peroxidase with the addition of hydrogen peroxide through the mechanism described, as illustrated in Scheme 1.

Biocatalytic systems based on HRP immobilized on nanomaterials have remarkable potential for polyphenol quantification in food samples and for purifying water and wastewater of a wide range of impurities, such as dyes, pharmaceuticals, pesticides, and phenols, which could be substrates of this enzyme [23]. The quantification of phenols based on the reduction of the products generated by the HRP reaction has advantages such as the simplicity of construction and handling; electrode protection against passivation due to the accumulation of secondary polymeric products on its surface; and the potential of working relatively low, avoiding possible interferences due to the oxidation of other compounds present in the sample matrix [24].

The biosensor we proposed combines the advantageous properties of Na^+^-Mt with those of GA-CHIT obtained through eco-friendly MR. As this polymer has not been previously used for this purpose, we evaluated different enzyme immobilization methodologies to achieve the best biosensor performance. The physicochemical characteristics of the Na^+^-Mt/GA-CHIT composite and the electrochemical response of polyphenols at glassy carbon electrodes modified with this composite were evaluated. The HRP immobilization method in the composite was carefully selected, and the response of the bioelectrode to different phenolic compounds was analyzed. As a proof of concept, the biosensor was applied to quantify the polyphenol content in pennyroyal and lemon verbena extracts.

## 2. Materials and Methods

### 2.1. Reagents

**Scheme 2:**
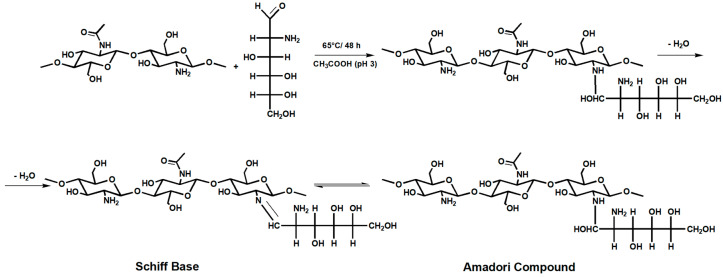
Chemical structure of GA-CHIT obtained by MR.

Sodium montmorillonite (Na^+^-Mt) (ion exchange capacity = 0.88 meq g^−1^) was from Cloisite Southern Clays (Southern Clay Co., Gonzales, TX, USA). Medium MW CHIT, HRP type II (EC 1.11.1.7 from horseradish) lyophilized powder containing 150–250 U mg^−1^ of solid and glutaraldehyde (GLUT) 25% *v*/*v* grade I were from Sigma-Aldrich (Merck-Sigma-Aldrich, Buenos Aires, Argentine). Hydroquinone (HQ), catechol (CAT), resorcinol (RES), chlorogenic acid (CGA), gallic acid (GaAc), hydrogen peroxide 30% *v*/*v*, Na_2_CO_3_, and Folin Ciocalteu (FC) reagent were from Biopack (Biopack, Buenos Aires, Argentine). Mono- and dibasic potassium phosphate were from Cicarelli (Cicarelli Laboratorios, Santa Fe, Argentine). The water-soluble glucosamine–chitosan derivative GA-CHIT was obtained by MR between CHIT and GA hydrochloride according to the procedure previously reported by Vanden Braber et al. [23]. The product obtained presents a deacetylation degree of (63 ± 1)%, a MW of (98 ± 15) kDa, and a solubility of (18 ± 2)% *w*/*w* or (1.38 ± 0.04) g L^−1^ in phosphate buffer, pH 7.40 and 25 °C. Scheme 2 shows the polycation chemical structure. Pennyroyal and lemon verbena leaves were obtained from a local market. All solutions were prepared with ultrapure water. All other chemicals were analytical reagent grade, and they were used without further purification. Ultrapure water (ρ = 18 MΩ cm) from a Millipore-MilliQ system was used for preparing all solutions. 

### 2.2. Preparation of Pennyroyal and Lemon Verbena Extracts

The extracts were obtained from 1.0 g of dry leaves in 10 mL of ultrapure water, heated to boiling temperature, with reflux and mechanical stirring, for 30 min. The extracts were filtered with Xinxing-grade 102 qualitative filter paper (medium filtering speed), and the corresponding absorbance spectra were recorded between 190 and 500 nm.

Determination of polyphenols by the Folin–Ciocalteu method: We followed the procedure described in the literature [25,26] with some modifications. Briefly, 3.15 mL of 20% *w/v* Na_2_CO_3_ and 650 µL of FC reagent were added to aliquots of a gallic acid solution or to the extracts, and the final volume was adjusted to 5.0 mL with ultrapure water to obtain standard concentrations between 0.005 and 0.03 mg mL^−1^. The solutions were placed in a thermostated bath at 50 °C for 10 min and then cooled to room temperature, and the absorbance was measured at 735 nm. 

### 2.3. Equipment

Fourier transform infrared (FTIR) spectra were recorded at room temperature with a Thermo Scientific Nicolet iN10 infrared microscope operating between 800 and 4000 cm^−1^ (64 scans; 4 cm^−1^ resolution); the spectrometer was equipped with an external reflection accessory (85 grazing angle). Solid powder of Na^+^-Mt and HRP was spread over a gold substrate, while Na^+^-Mt/GA-CHIT dispersion was deposited on a gold-covered glass slide and allowed to dry at 60 °C. A pre-cleaned gold-covered glass slide was used as a reference. X-ray powder diffraction (pXRD) experiments were performed with a PANalitycal X’Pert Pro diffractometer using Bragg–Brentano geometry, operated at 40 kV and 40 mA, with Cu Kα radiation (λ = 1.5418 Å), equipped with a pixel 1D detector with 255 channels. X-ray diffraction patterns were obtained between 5.0 and 30.0 in 2θ with an optical pitch of 0.0260. The patterns were obtained for Na^+^-Mt and GA-CHIT as powder, while the water dispersion of the Na^+^-Mt/GA-CHIT composite (mass ratio 20:1) and the Na^+^-Mt were deposited over a glass slide and allowed to dry at 60 °C. Zeta potential (ζ) was measured at 25 °C with a nanoparticle size analyzer (SZ-100, Horiba Scientific, Kyoto, Japan) equipped with a laser diode of 532 nm and 10 mW of power. An average value of fifteen measurements was used. Cyclic voltammetry (CV) and chronoamperometric (CA) experiments were carried out with a potentiostat–galvanostat Teq4 (NanoTeq, Buenos Aires, Argentina). The experiments were performed in a three-compartment electrochemical cell. A large-area platinum wire and an Ag/AgCl/3 M NaCl electrode (Model RE-5B, BAS, Brooklyn, NY, USA) were used as counter and reference electrodes, respectively. The reported potentials are referred to later. Glassy carbon electrodes (GCE) with 0.071 cm^2^ of geometric area (Model CHI104, CH Instruments, Tennison Hill Drive Austin, TX, USA) were used as working electrodes. 

### 2.4. Electrochemical Measurement Procedures and Statistical Analysis of the Response

The electrochemical response of polyphenols was analyzed in a 0.10 M, pH 5.0 phosphate solution. Solutions were deoxygenated by controlled N_2_-bubbling 20 min prior to measurements, and the gas flow was maintained over the solution during the experiments. CA experiments were carried out at −0.200 V under convective conditions, achieved by magnetic stirring. The transient current was allowed to decay to a steady state value before adding a given amount of analyte, and subsequently generated current was monitored over time to obtain calibration curves. The sensitivity, S, of the biosensors was determined from the slope of the fitting line of the linear portion of the calibration curve. The detection limit (LOD) and quantification limit (LOQ) reported are, respectively, the concentration of the analyte that produces a signal 3.3 and 10 times greater than the standard deviation of the blank (sd) divided by S. The LOQ value was taken as the lower limit of the linear range, while the analyte concentration value from which the calibration curve deviates from linear behavior was taken as the upper limit. The response time was the time required to reach 90% of the stationary current obtained when the analyte was added. Repeatability was assessed using the coefficient of variation (VC) of the sensitivity of the bioelectrodes obtained in exactly the same way, measured by the same operator, under identical environmental conditions, using the same device, over a short period of time. VC values were determined as a ratio between the standard deviation and the mean of the sensitivity values [27].

The concentration of polyphenols in pennyroyal and lemon verbena samples was determined by the standard addition method. Before sample addition, aliquots of a CGA standard solution were added in order to confirm the current response and concentration proportionality. The reported values are the average of 3 independent measurements.

### 2.5. Preparation of Na^+^-Mt/GA-Chit Composite

First, dispersions of Na^+^-Mt and GA-CHIT were separately prepared in deionized water and mechanically stirred for 24 h. Then, equal volumes of Na^+^-Mt (4.0 mg mL^−1^) and GA-CHIT (1.0 mg mL^−1^ or 0.2 mg mL^−1^) were mixed, adding the polycation dropwise to the Na^+^-Mt suspension. In both cases (Na^+^-Mt 4 mgmL^−1^ + GA-CHIT 1.0 mg mL^−1^, mass ratio 4:1; Na^+^-Mt 4 mg mL^−1^ + GA-CHIT 0.2 mg mL^−1^, mass ratio 20:1), the mixtures were mechanically stirred for 24 h and then centrifuged at 10,000 rpm for 15 min to separate the precipitate (composite) from the supernatant, which could contain an excess polycation. The pellets were suspended in 1.0 mL of deionized water. The procedure is shown in Scheme S1 in the SI. 

### 2.6. Surface Modifications and Construction of the Bioelectrode

The GCE was polished to a mirror-like finish with 0.05 µm alumina powder for 2 min; rinsed thoroughly with deionized water; and sonicated for 30 s in water, successively. Then, the electrode was cycled between −0.300 V and 0.800 V at 0.100 Vs^−1^ for 15 cycles in 0.10 M, pH 7.0 phosphate buffer solution. The electrode surface was modified by adding 15 µL of the composite Na^+^-Mt/GA-CHIT, which was left at room temperature for 2 h before incorporating the HRP enzyme.

## 3. Results and Discussion

### 3.1. Characterization of Na^+^-Mt/GA-CHIT Composite 

**Figure 1:**
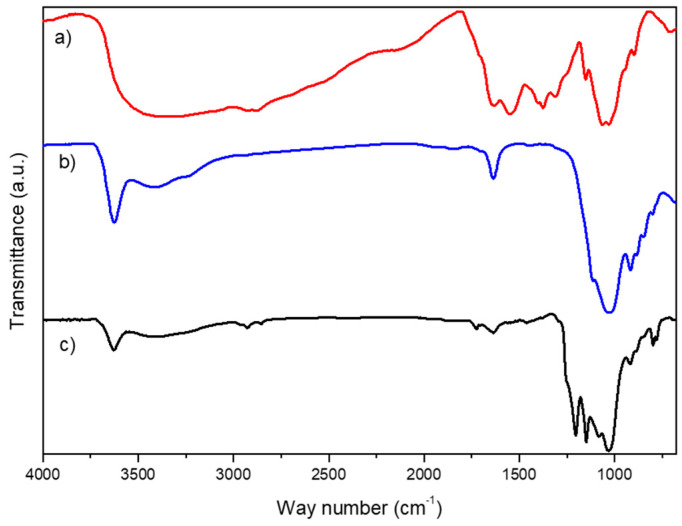
FTIR spectra of GA-CHIT dispersion (a), Na^+^-Mt (b), and Na^+^-Mt/GA-CHIT composite dispersion (mass ratio 20:1) (c).

The characterization of the composite in the absence of the enzyme was achieved by FTIR spectroscopy, as well as pXRD, to determine the type of interaction (exfoliation, intercalation, or two-phase formation) between the clay and the polycation. Figure 1 shows the FTIR spectra of the GA-CHIT and Na^+^-Mt/GA-CHIT composite dispersed in water, deposited, and dried over a gold substrate (see Section 2.2), together with the spectra of solid Na^+^-Mt. The spectra of the polycation show a strong and wide band in the area between 3500 and 3400 cm^−1^ as a result of the −OH stretching vibration associated with free, inter-, and intra-molecular hydroxyl groups, superimposed with the N-H stretching of the primary amine. Another characteristic band appears at 2950 cm^−1^ and 2860 cm^−1^, attributable to the antisymmetric and symmetric stretching of the methyl groups, respectively. A clear signal at 1653 cm^−1^ corresponding to the amide I band (stretching vibration of the acetamido group) can be observed together with signals at 1586 cm^−1^ and 1355 cm^−1^ corresponding to amide II and an amide III band, respectively. The bands at 1400 and 1375 cm^−1^ can be attributed to the bending vibrations of the H-C-H bonds of the methylene and methyl groups, respectively. The signal appearing at 1150 cm^−1^ is due to the antisymmetric stretching of the C-O-C bridge, and those at 1060 cm^−1^ and 1030 cm^−1^ can be attributed to the elongation of the C-O bond [26]. The Na^+^-Mt spectra exhibit signals at 3600 cm^−1^ due to the stretching vibration of the O-H bond; a band in the region of 3400–3200 cm^−1^ as a result of interlayer and intralayer H-bonded and O–H stretching [28]; a strong signal at 1636 cm^−1^ due to the bending H-O-H, as well as a signal at 1030 cm^−1^ from the Si-O and Si-O-Si stretching bonds; and bands of AlAlOH-bending vibrations at 912 cm^−1^ and AlMgOH around 840 cm^−1^ [29]. Other characteristic bending vibrations in Si–O bonds typically observed at 692 cm^−1^ and 529 cm^−1^ were outside of the analyzed range. The spectrum of the Na^+^-Mt/GA-CHIT composite (mass ratio 20:1) shows a combination of bands corresponding to both components, the polymer and the clay, indicating the successful assembly of Na^+^-Mt and GA-CHIT. Additionally, an intense peak at 1725 cm^−1^, attributable to the axial deformation of the C=O of amide I, can be observed [30], along with a shift toward 1560 cm^−1^ of the location of the amide II band, indicating that the –NH_2_ group is involved in the interaction with the clay. The possible interaction could be the hydrogen bonding (–OH…NH_2_–) between the –NH_2_ group in GA-CHIT and –OH on the edge of Mt [31,32].

**Figure 2:**
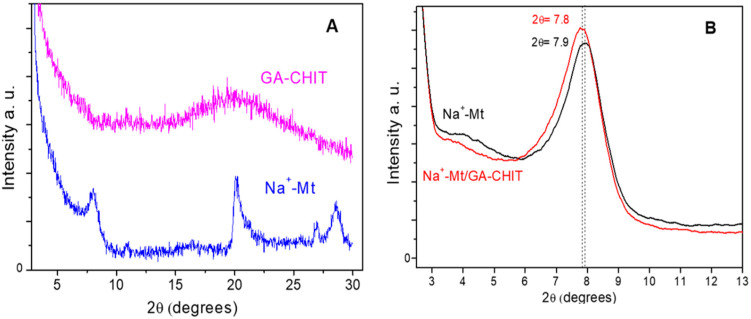
pXRD patterns of (**A**) GA-CHIT and Na^+^-Mt and (**B**) Na^+^-Mt/GA-CHIT composite (mass ratio 20:1) and Na^+^-Mt water dispersion deposited and dried. Dashed lines in (**B**) indicate the value of 2θ.

Figure 2A shows the XRD pattern of GA-CHIT and Na^+^-Mt. For the polycation, a broad peak centered at 2θ = 21.00 can be observed, indicating the existence of an amorphous structure [33]. The Na^+^-Mt pattern exhibits a strong signal at 2θ = 7.90, corresponding to basal spacing (d001), and, additionally, signals at 20.10 and 28.60 belonging to the planes oriented in 100 and 005, respectively, in the crystal structure of Na^+^-Mt [34]. It has been reported that the movement of the basal reflection (d001) of Na^+^-Mt toward a lower angle indicates the formation of an intercalated nanostructure [35], while peak broadening and intensity decreases most likely indicate a disordered intercalated or exfoliated structure. The complete disappearance of this signal indicates complete clay exfoliation [36].

To evaluate the possible interactions of GA-CHIT with the clay, we analyzed the XRD pattern of Na^+^-Mt and the composite Na^+^-Mt/GA-CHIT dispersed in water, which was deposited and dried over a glass substrate (Figure 2B). Reflection peaks at 2θ = 7.90 (d001 = 1.12 nm) and 7.80 (d001 = 1.13 nm) can be observed for the clay and the composite Na^+^-Mt/GA-CHIT, respectively. These values are characteristic of one layer of H_2_O molecules within the interlaminar space of Na^+^-Mt [37] and indicate that, under the conditions of the composite’s preparation, GA-CHIT is not intercalated between the layers of Na^+^-Mt and that the laminar structure of the clay is preserved; that is, no exfoliation occurred. Therefore, the composite is formed by a mixture of the two phases favored by an electrostatic interaction between the negatively charged silicate layers and the positively charged GA-CHIT or/and the formation of H-bonds with the end face -OH groups of the clay. Previous reports have shown that exfoliation, intercalation, two-phase formation (with CHIT deposited on stacked clay sheets), and their combinations depend on the experimental conditions used to prepare the composite, such as the mass ratio of Na^+^-Mt/CHIT [38], the molecular weight of CHIT [39], and the temperature [32]. The excess of polycation favors exfoliation, but as the Na^+^-Mt content increases, the coexistence of both intercalated (Na^+^-Mt and CHIT multilayer stacks) and exfoliated structures can be observed. By increasing the Na^+^-Mt content up to 10 wt %, an intercalated morphology with occasional flocculation can be seen [36]. 

The advantages or disadvantages of each type of structural arrangement must be analyzed considering the intended purpose, the immobilization of the positively charged HRP. Although, in the interlayered composite, the gaps between the clay layers increase, the negative charges of Na^+^-Mt required for the electrostatic interaction with HRP are neutralized, or excess positive charges can be reached, which is unfavorable for the physisorption of HRP due to its isoelectric point (between 8.7 and 9.0 [22]). On the other hand, the advantage of intercalated clays is the facilitated diffusion of the substrate and oxygen as a co-substrate of the enzymatic reaction to the electrode surface. In the exfoliated composite, Na^+^-Mt sheets are completely separated and surrounded by an excess of GA-CHIT. Again, these characteristics are unfavorable for enzyme adsorption due to electrostatic repulsion. On the other hand, for effective enzyme covalent binding, it is necessary for the functional groups of GA-CHIT to be accessible to the cross-linking agent, while avoiding cross-linking between polymer chains to facilitate subsequent enzyme binding at activated sites. The mass ratio selected for our experiments, with a high proportion of Na^+^-Mt, favors the formation of a two-phase organization with the polycation deposited on the stacked clay sheets. However, the colloidal dispersion remained stable over time due to the particle’s charge. Furthermore, the negative charge density of the composite would allow the approach of the positively charged enzyme, favoring its adsorption through van der Waals forces, electrostatic interactions, hydrogen bonds, and hydrophobic interactions for the non-covalent immobilization of the protein. Additionally, the functional groups of GA-CHI on the surface of stacked Na^+^-Mt sheets would allow for the covalent immobilization of HRP through a cross-linking agent such as glutaraldehyde [17]. This adsorption method requires the enzyme to approach the nanomaterial surface before the covalent bond formation. In this sense, the negative charges of the composite would favor this step. Additionally, the exposure of GA-CHIT could be more favorable than in intercalated or exfoliated composites since intermolecular cross-links could occur in these last cases.

### 3.2. Electrochemical Behavior of Polyphenols at GCE Modified with the Na^+^-Mt/CHIT Composite

According to Scheme 1, the electrochemical detection of polyphenols is based on the reduction, at a certain potential, of the radicals formed during the catalytic reaction of HRP at the surface of the GCE modified with the composite. Therefore, the sensitivity of the biosensor depends on the electrochemical response of the polyphenols on the GCE/Na^+^-Mt/GA-CHIT. The electrochemical behavior of HQ, CAT, and RES was analyzed by CV in a potential range of −0.300 V to 1.00 V in a solution of 0.10 M of KH_2_PO_4_, pH 5.0.

**Figure 3:**
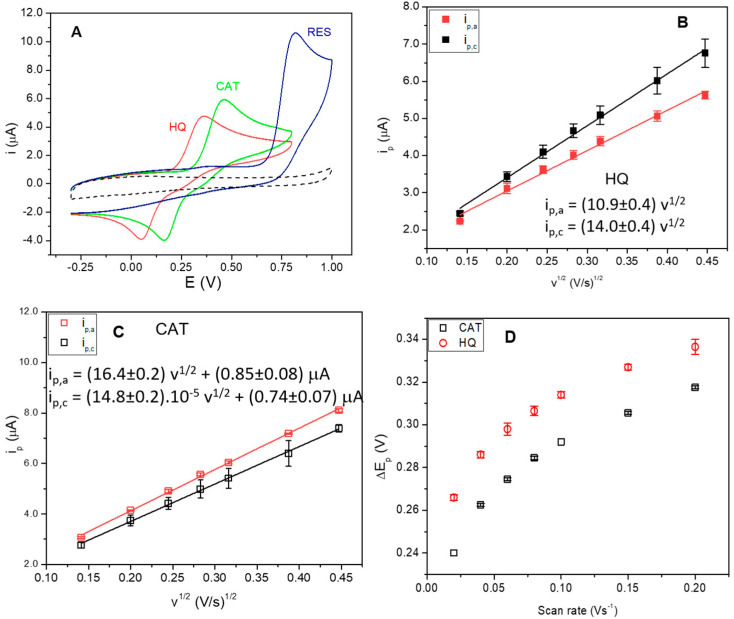
Cyclic voltammograms of the supporting electrolyte (dashed line) and 20 µM solutions of HQ, CAT, and RES at GCE/Na^+^-Mt/GA-CHIT at 0.100 Vs^−1^ (**A**). Anodic (i_pa_) and cathodic (i_pc_) peak currents of HQ (**B**) and CAT (**C**) as functions of v^1/2^. ΔE_p_ as a function of scan rate (**D**). The values are the average of three independent measurements. Supporting electrolyte: 0.10 M KH_2_PO_4_ pH 5.0.

Figure 3A shows the voltamperograms recorded in 20 µM solutions of HQ, CAT, and RES at 0.100 Vs^−1^. A pair of quasi-reversible redox peaks can be observed for HQ and CAT, while irreversible oxidation is produced for RES, indicating that all the electroactive substances can reach and be detected at the electrode surface. The influence of the potential scan rate on the electrochemical response of HQ and CAT was also evaluated, and the results obtained are shown in Figure 3B,C. The peak currents (i_p_) increased with the scan rate in the studied range, remaining linear with the square root of the scan rate (v^1/2^), indicating that the responses are diffusion-controlled processes. As expected, for both molecules, an increase in the peak potential separation (ΔE_p_) with the scan rate can be observed (Figure 3D). According to the obtained results, −0.200 V is an appropriate potential for the electrochemical detection of the radicals formed during the enzymatic reaction.

### 3.3. Selection of the Composite Proportion and HRP Adsorption Methodology

After verifying the appropriate electrochemical response of the phenolic compounds, it is necessary to select a suitable ratio of Na^+^-Mt/GA-CHIT to modify the GCE and adsorb the HRP enzyme. For this purpose, we compared the analytical responses of biosensors built through the physisorption of HRP on top of GCE/Na^+^-Mt/GA-CHIT obtained with composites prepared with a clay–polycation mass ratio of 4:1 and 20:1. The response to the addition of HQ was evaluated by measuring the steady currents at −0.200 V in the presence of H_2_O_2_. The sensitivity and reusability of the biosensor were analyzed. The average values of sensitivity of the three independent measurements; the coefficient of variation of the sensitivity; and the linear range were (0.220 ± 0.002) mA mM^−1^, 9%, (5–15) µM and (0.190 ± 0.001) mA mM^−1^, 5%, (1–9) µM for the 4:1 and 20:1 composites, respectively. Both composites were used five days after their preparation to modify electrodes and to adsorb HRP from the fresh protein solution. For the composite with the Na^+^-Mt/GA-CHIT 4:1 ratio, a decrease of 40% in the sensitivity compared with that obtained with the recently prepared composite was determined, while no significant differences were observed with the 20:1 ratio (Figure S1 in the SI). This indicates that the composite with the highest proportion of polycation is unstable over time, thus requiring new preparation each time. These results can be rationalized considering the isoelectric point of HRP and the charge of the Na^+^-Mt/GA-CHIT composite. According to our results, Table 1, for the composite with a mass ratio of 4:1, a decrease in the ζ of Na^+^-Mt can be observed, probably as a consequence of the positive charges of the polymer on the surface of the clay sheets. On the other hand, this effect does not occur for the 20:1 composite due to the low proportion of polymer in the mixture, which is favorable for the electrostatic interaction with HRP that is positively charged at the working pH.

Given these results, a mass ratio of 20:1 was selected for further experimentation. Considering the time and costs required preparing the bioelectrode, the stability of the response upon subsequent use was evaluated. For the selected composite, the sensitivity decreases after the first use by around 45 % (Table S1 in the SI). This fact could be associated either with desorption of the protein or with the loss of enzymatic activity. In the literature, pristine clays have been used as immobilization matrices for biomolecules by adsorption onto them due to the opposite charges of clays and enzymes. The exchangeable cations can be replaced by positive enzyme molecules when the pH of the enzyme solution is lower than the isoelectric point of the protein, as in the case of HRP. In addition, the deprotonation of hydroxyl groups on the surface of clay minerals leads to variable negatively charged sites at the edges of clay minerals where the positive enzymes can also adsorb through electrostatic interactions. In addition to electrostatic interactions, hydrophobic interactions, hydrogen bonds, and salt bridges may also be involved in the binding of enzymes to clay minerals. However, the stability of the enzyme is low, and it can detach from the clay surface as a result of very small environmental changes (pH, ionic strength, concentration of biomolecules, etc.) [40]. Accordingly, such leaching of enzymes in a liquid-phase system gives rise to a rapid loss of the activity of clay mineral enzyme composites as catalysts or biosensors. Consequently, although the immobilization of enzymes in clay minerals by non-covalent adsorption is a simple and ecological route, the interactions are weak compared with covalent bonds and do not maintain a stable response or enable the reuse of the bioelectrode. For this reason, the covalent bonding of the enzyme on the electrode modified with the composite was analyzed. The immobilization of HRP was carried out by covalent bonding using GLUT to cross-link the -NH_2_ groups of HRP and GA-CHIT and improve the protein retention in the composite. GLUT has been broadly practiced for protein immobilization using different support surfaces [21,41], as it possesses two aldehyde groups that can covalently connect to other substances via either an aldol reaction or a Schiff base reaction. For example, Luo et al. used an amine-rich chitosan polysaccharide hydrogel for horseradish peroxidase immobilization through gold nanoparticle self-assembly [42]. HRP was also immobilized covalently by using GLUT on reduced graphene oxide and applied to the biodegradation of phenols [43]. Different carbon nanostructure matrices have also been used as supports for the covalent union of HRP [24].

The response of the biosensor prepared with different strategies was compared in order to select the method that obtained the highest sensitivity and the lowest loss of response: (a) the activation of the amine groups exposed on the surface of GCE/Na^+^-Mt/GA-CHI with a 0.125% *v*/*v* GLUT solution prior to HRP adsorption over Na^+^-Mt/GA-CHIT/GLUT/HRP; (b) the deposition of a mixture containing 0.125% *v*/*v* of GLUT and 0.1 mg mL^−1^ of GA-CHIT over GCE/Na^+^-Mt/GA-CHIT/HRP_phys_; (c) the cross-linking of the physisorbed enzyme at GCE/Na^+^-Mt/GA-CHIT/HRP_phys_; and activation of the amino groups available to GCE/Na^+^-Mt/GA-CHIT with a GLUT solution of 0.125% *v*/*v* (d_1_) or 0.250% *v*/*v* (d_2_) before HRP adsorption (Scheme S2 in the SI).

**Figure 4:**
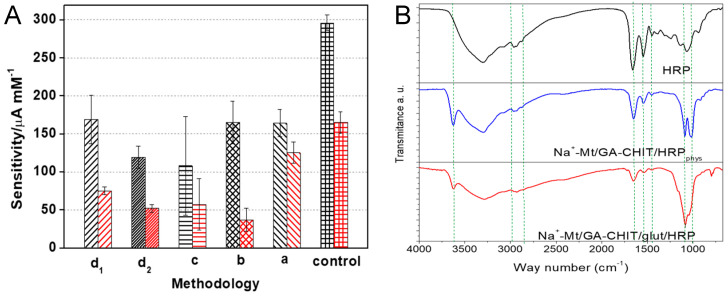
(**A**) Sensitivity to HQ for the first (black bars) and second use (and red bars) of GCE/Na^+^-Mt/GA-CHIT/HRP biosensors prepared with the protocols described in the text and shown in Scheme S2. The values corresponding to physisorbed HRP without GLUT are also included as a control. The values are the average of three independent measurements. Experimental conditions: E_ap_ = −0.200 V; supporting electrolyte: H_2_O_2_ 600 µM in KH_2_PO_4_ 0.10 M pH 5.0. (**B**) FTIR spectra of HRP, Na^+^-Mt/GA-CHIT/HRP_phys_, and Na^+^-Mt/GA-CHIT/GLUT/HRP.

Figure 4A shows the sensitivity for the first and second use of the biosensor and the response obtained with physisorbed HRP in the absence of GLUT as a control experiment. The smallest loss of sensitivity due to a second use of the biosensor (25%) was obtained with methodology (a), in which the exposed functional groups of GA-CHIT on the surface of the composite were activated to allow for the covalent binding of HRP. As reported, the immobilization of enzymes on supports previously activated with GLUT is carried out in two steps; the first involves the electrostatic adsorption of the enzyme, while the second is the covalent bond formation between the nucleophilic groups of the physisorbed protein and the aldehyde groups of GLUT on the support [44]. Through this procedure, bonds are formed mainly with the exposed terminal -NH_2_ groups of the protein, reducing the impact on the possible conformational changes [45]. In our case, methodology (a) allows for the physisorption of HRP, favored by the charge of the composite on the electrode surface and the low ionic strength of the enzyme solution, as well as the formation of covalent bonds with exposed amine groups, preventing GLUT molecules from reaching the internal regions of the protein structure [46]. At difference, in methodologies (b) and (c) covalent bonds can be established with the internal functional groups of the protein, producing conformational changes and leading to a decrease in sensitivity of 75% and 46%, respectively. Methodology (d) leads to an average loss of response of 56% due to the internal cross-linking of GA-CHIT, probably leaving few activated sites for HRP binding. From the results obtained, it can be claimed that the most appropriate method is (a) because, even though the sensitivity of the biosensor is lower than that of the control experiment, covalent immobilization allows for its reuse with a significantly smaller decrease in the response. On the other hand, it has been reported that during surface activation with GLUT, dimers can be generated, which react more easily with the enzyme [44,46]. 

As a control for the protein immobilization process, FTIR spectra were analyzed; Figure 4B shows the FTIR spectra of the HRP air-dried on the support; physisorbed into the composite, Na^+^-Mt/GA-CHIT/HRP_phys_; and covalently linked to the composite through GLUT, Na^+^-Mt/GA-CHIT/GLUT/HRP. HRP shows a typical protein spectrum, with amide I, II, and III bands at 1660 cm^−1^, 1546 cm^−1^, and 1244 cm^−1^, respectively. The absorption bands between 1350 cm^−1^ and 1450 cm^−1^ can be attributed to the heme mode vibration [47]. Additionally, signals corresponding to the stretching of the O-H bond and the antisymmetric and symmetric stretching of the methyl groups can also be observed, the latter located between 2960 cm^−1^ and 2870 cm^−1^. The spectra of the enzyme physisorbed or covalently linked to the composite show signals corresponding to the contribution of the composite (see Figure 1) and the protein. This evidence indicates the successful immobilization of the biocatalyst on the support surfaces. Furthermore, no substantial differences between any of spectra can be observed, suggesting that no drastic changes in the secondary structure occurred as a consequence of the immobilization processes. This result is in agreement with previous reports where HRP has been successfully cross-linked to CHIT without significant conformational changes in the protein structure [24,48].

The stability of the bioelectrode prepared with methodology (a) was analyzed after successive uses and over time. The bioelectrode retains 75% of its initial response to HQ after six successive uses and maintains 37% of its initial response after 8 days, keeping the electrode at 8 °C between uses (Figure S3).

### 3.4. Chronoamperometric Response of Phenolic Compounds

**Figure 5:**
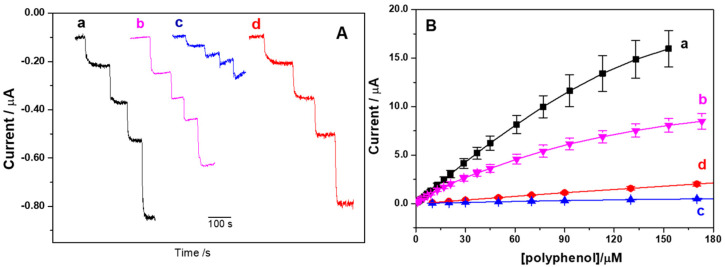
(**A**) Amperometric response of HQ (a), CGA (b), RES (c), and CAT (d) at GCE/Na^+^-Mt/GA-Quit/GLUT/HRP. E_ap_ = −0.200 V; supporting electrolyte: H_2_O_2_, 600 µM in KH_2_PO_4_, 0.10 M, pH 5.0. Current responses for 1, 2, 3, and 5 µM, (a) and (b); 10, 20, 30, and 50 µM, (c) and (d), for the respective polyphenols. (**B**) Calibration plots for HQ (a), CGA (b), RES (c), and CAT (d). The values are the average of three independent experiments.

The biosensor prepared under the selected conditions was used to evaluate the amperometric response of HQ, RES, and CAT. The response to CGA was also characterized, which will be used as a standard in phenolic compound determination in plant extract samples using the HRP biosensor. Polyphenols were added to a solution of 0.10 M of KH_2_PO_4_, pH 5.0, containing 600 µM of H_2_O_2_, and current–time variations at −0.200 V were recorded. Figure 5 illustrates stationary currents reached after increasing concentrations of (a) HQ, (b) CGA, (c) RES, and (d) CAT. From the corresponding calibration plots, Figure 5B, the analytical parameters were estimated for each phenolic compound (Table 2). Blank experiments performed on GCE/Mt/GA-CHIT (Figure S2 in the SI) demonstrate that the current signals obtained were solely due to the electrochemical reduction at the applied potential of the oxidized polyphenols generated during the enzyme catalytic reaction.

Table 2 collects the average values of the electroanalytical parameters obtained for the developed biosensor and compares them with those reported for others containing HRP regarding HQ, CGA, CAT, and RES. Even though the average concentration of polyphenolic compounds in plant extracts can vary significantly depending on several factors (plant species, part of the plant used, extraction method, and plant growth conditions, among others), the results obtained demonstrate the excellent performance of our bioelectrode, considering values previously reported for yerba mate and green coffee extracts, 13.2 and 0.04 μM, respectively [49].

**Table 2:** Electroanalytical properties of HRP biosensors regarding HQ, CGA, CAT, and RES.

Substrate	Sensitivity (μA mM^−1^)	LOD *	Linear Range (μM)	Time of Response (s)	Reference
HQ	(1.6 ± 0.0) × 10^2^	(74 ± 8) nM	0.22–29	12 ± 1	This work
HQ	~2.5 **	1.26 μM	5.0–30	-	[50]
HQ	15.32	0.6 μM	1.6–15	-	[51]
HQ	218 ± 2	1.6 nM	up to 120	27 ± 3	[20]
HQ	8	6.42 μM	16–240	2	[43]
CGA	(1.2 ± 0.1) × 10^2^	(26 ± 3) nM	0.08–9	9 ± 1	This work
CGA	132	2.7 nM	up to 4.2	-	[20]
CGA	22.7	0.7 μM	1–50	-	[45]
CAT	(16 ± 2)	(0.74 ± 0.09) μM	2.2–210	14 ± 1	This work
CAT	0.28	0.16 μM	20–32.5	-	[49]
CAT	18	0.22 μM	0.5–8	-	[51]
CAT	2	0.93 μM	1.6–8	2	[52]
RES	(3.7 ± 0.3)	(3.3 ± 0.2) μM	9.9–70	11 ± 1	This work
RES	16	-	-	-	[53]

* LOD: detection limit; ** estimated from reported calibration curve.

The differences in sensitivity values obtained for the three isomers, HQ, CAT, and RES, could be explained in terms of the -OH positions in the ring that act as an e-donating group, contributing to increasing the electronic density of the π system of the aromatic ring and favoring the stabilization of the radicals generated during enzyme reduction [54]. However, the resonance stabilization effect is greater in the CAT and HQ molecules since the -OH groups are found in ortho- (o) and para- (p) positions with each other, respectively. As the stability of the radicals formed increases, a greater sensitivity is obtained by the biosensor. For its part, CGA has an additional substituent group, derived from acrylic acid, which enables the delocalization of the unpaired electrons of the free radical and would explain the higher sensitivity obtained compared with that of CAT. 

Other substances present in the samples that can undergo an electrochemical reaction on the electrode surface at the applied potential and that are not the enzyme substrate could interfere with the biosensor’s response. For example, ascorbic acid (AA) can be electrochemically oxidized at low potential and/or react with added H_2_O_2_, decreasing the biosensor response. Tartaric acid (TA), glutamic acid (GlA), citric acid (CA), and glucose (Gluc) can also affect the current response [47]. To evaluate the possible interference, the relative intensity changes in the current of HQ after the addition of TA, GlA, CA, Gluc, and AA were evaluated. Two proportions of HQ–interferent, 1:2 and 1:4, were tested. No significant interference was found for the first four compounds, while for AA, decreases in the responses of 2.5% and 5% for 1:2 and 1:4 HQ/AA proportions, respectively, were observed.

The Michaelis–Menten constant (K_m_) is an important parameter in enzyme kinetics. It indicates the relative affinity of an enzyme for its substrate. More specifically, it is the substrate concentration at which the reaction rate reaches half its maximum value. The lower its value, greater is the affinity of the enzyme for its substrate, and vice versa [48]. According to the Eadie–Hofstee plots (i vs. i/[substrate]), the apparent Michaelis–Menten constants K’_m_ for immobilized HRP were calculated (Figure S4 in the SI). The values obtained for HQ, CAT, and RES were (0.42 ± 0.05) mM, (2.20 ± 0.07) mM, and (0.29 ± 0.02) mM, respectively. These values are in the range of values (0.11–3.2 mM) previously reported by other authors [49], ensuring that the immobilized enzyme retains its essential catalytic properties. In the case of CGA, the Eadie–Hofstee plot shows two well-defined slopes. Under normal conditions, an enzyme with a single active site, such as HRP, should not have two Km values for a given substrate. However, cases may arise where experimental data show discrepancies, as in the case of CGA (9 ± 2) µM and (80 ± 10) µM. This could be the result of several factors, such as an inhibition effect, the aggregation of the enzyme or substrate, or the existence of two enzyme populations, with different accessibility for the active site [55].

This behavior could also be due to the existence of sites with different accessibility to the enzyme, one with higher and another with lower affinity for the substrate.

### 3.5. Determination of Polyphenols in Pennyroyal and Lemon Verbena Plant Extracts

**Figure 6:**
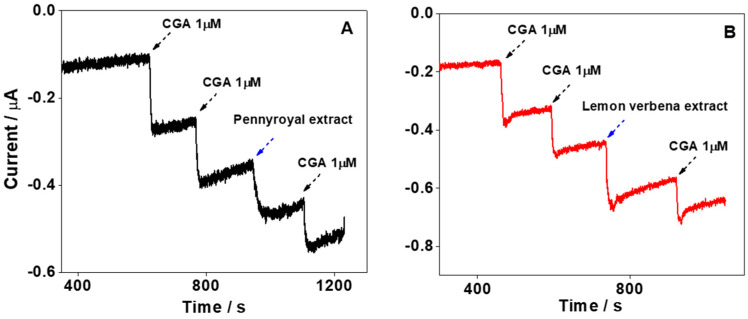
CA response of the optimized bioelectrode to additions of CGA, 1 μM, and pennyroyal (**A**) or lemon verbena extracts (**B**) (as indicated in the figure). Supporting electrolyte: H_2_O_2_, 600 µM in 0.10 M of KH_2_PO_4_ at pH 5.0. E_ap_ = −0.200 V.

The polyphenol concentrations in the extracts of pennyroyal and lemon verbena were determined using a GCE/Na^+^-Mt/GA-CHIT/GLUT/HRP bioelectrode. Figure 6 shows the amperometric recording obtained with the standard addition method, using GCA. The results obtained were (3.1 ± 0.2) mmol L^−1^ and (2.16 ± 0.03) mmol L^−1^ for the pennyroyal and lemon verbena samples, respectively. In order to validate these results, they were compared with those obtained with the FC method. The total contents of polyphenols (TCPs) in the samples, determined by this method, were (3.2 ± 0.4) mmol L^−1^ for pennyroyal and (2.35 ± 0.06) mmol L^−1^ for lemon verbena. In the case of pennyroyal, no significant differences were found between the values obtained using the biosensor and the FC method. However, for lemon verbena, the TCP was higher when the latter was used. As reported in previous studies, TCPs determined by this method are higher than those obtained using HRP biosensors [47]. These differences could be attributed to the lack of selectivity of the FC method toward polyphenols, with various substances that can participate in the redox reaction involved, such as sugars (glucose and fructose), proteins, organic acids, and Fe^2+^ ions, among others. 

In this context, it is important to highlight the advantages of using electrochemical biosensors given their higher selectivity and specificity; shorter analysis and sample preparation time; lower sample volume; and less expensive equipment. Despite the enzymatic costs, HRP-based enzymatic biosensors have been reported for the determination of the polyphenol content of yerba mate and green coffee (20), as well as in environmental samples (wastewater from coffee washing) [51], antioxidants in food matrices [56], and endocrine-disrupting compounds in pharmacological products [57].

## 4. Conclusions

In accordance with the proposed objective of this work, we demonstrated for the first time the advantages of using glucosamine–chitosan synthesized by the Maillard reaction associated with sodium montmorillonite to obtain a nanohybrid composite to immobilize horseradish peroxidase enzyme and develop an electrochemical biosensor for polyphenol quantification. The developed biosensor combines the advantageous properties of clay with those of the chitosan derivative, such as improved water solubility, reduced molecular weight and viscosity, and eco-friendly synthesis, to obtain a composite with good adhesiveness on the transductor surface and a capacity for enzyme adsorption. The evidence obtained by infrared spectroscopy, X-ray diffraction, and Zeta potential indicates that the nanohybrid material is composed of two phases, with the polycation deposited on the stacked clay sheets, and exhibits a negative charge density. These characteristics are associated with the high proportion of clay–polycation used and favor the approach of positively charging HRP to immobilize them at the surface of the modified electrodes. The covalent immobilization of the HRP in the composite through the functional groups of GA-CHIT allowed us to obtain an electrochemical enzymatic biosensor with acceptable stability for successive uses and whose analytical parameters are suitable for polyphenol quantification in pennyroyal and lemon verbena extracts. The sensitivity, LOD, and time of response values obtained with the biosensor for hydroquinone, chlorogenic acid, catechol, and resorcinol are (1.6 ± 0.2) × 10^2^ µA mM^−1^, (74 ± 8) nM and 12 s; (1.2 ± 0.1) × 10^2^ µA mM^−1^ (26 ± 3) nM and 9 s; (16 ± 2) µA mM^−1^ (0.74 ± 0.09) μM and 14 s; and (3.7± 0.3) µA mM^−1^ (3.3 ± 0.2) μM and 11 s, respectively. The results obtained demonstrate the excellent performance of our bioelectrode considering values previously reported in the literature for polyphenols in samples such as yerba mate and green coffee extracts, meaning the biosensor represents an alternative or complementary method for the determination of antioxidants in beverages. In addition, it combines relatively low-cost and environmentally sustainable materials, adding a new use for this type of chitosan derivative, which is usually synthesized for other purposes.

## Figures and Tables

**Table 1:** Zeta potential of Na^+^-Mt, GA-CHIT, and Na^+^-Mt/GA-CHIT composite with mass ratios of 20:1 and 4:1.

Sample	Zeta Potential (mV) *	
Na^+^-Mt	(−86 ± 2)	
GA-CHIT	(+39 ± 2)	
Na^+^-Mt/GA-CHIT composite	Mass ratio, 4:1	Mass ratio, 20:1
	(−69 ± 4)	(−87 ± 4)

* *n* = 15.

## Data Availability

The data will be made available upon request.

## Supplementary Materials

The following supporting information can be downloaded at https://www.mdpi.com/article/10.3390/bios14060278/s1: Scheme S1: Procedure to prepare Na^+^-Mt/GA-CHIT composite. Figure S1: Sensitivity of the bioelectrodes as a function of Na^+^-Mt/GA-CHIT mass ratio. Table S1: Sensitivity of GCE/Na^+^-MT/GA-CHIT/HRP as a function of the composite’s aging. Scheme S2: Methodology used to immobilize HRP with GLUT. Figure S2: Black experiments for CAT, RES, and CGA. Figure S3: Variation in the bioelectrode response for successive uses and over time. Figure S4: Eadie–Hofstee plots for HQ, CAT, RES, and CGA.
