# Supplementary material for: Immobilization of Horseradish Peroxidase onto Montmorillonite/Glucosamine–Chitosan Composite for Electrochemical Biosensing of Polyphenols
Source: Biosensors (Basel). 2024 May 29;14(6):278. doi: 10.3390/bios14060278 (PMC11201438; doi:10.3390/bios14060278)
Supplement: Supplementary file 1 [file biosensors-14-00278-s001.zip › biosensors-2965153-supplementary.pdf]

biosensors

MDPI

Article

Immobilization of Horseradish Peroxidase onto Montmorillonite/Glucosamine–Chitosan Composite for Electrochemical Biosensing of Polyphenols

María Belén Piccoli [1], Florencia Alejandra Gulotta [2], Mariana Angélica Montenegro [3], Noelia Luciana Vanden Braber [3], Verónica Irene Paz Zanini [4,*] and Nancy Fabiana Ferreyra [1,*]

1 Instituto de Investigaciones en Fisicoquímica de Córdoba (INFIQC-UNC-CONICET), Departamento de Fisicoquímica, Facultad de Ciencias Químicas, Universidad Nacional de Córdoba, Córdoba X5000HUA, Argentina; belen.piccoli@unc.edu.ar
2 Independent Researcher, Santiago del Estero G4206XCP, Argentina; florgulotta@gmail.com
3 Centro de Investigaciones y Transferencia de Villa María (CIT-VM), Consejo Nacional de Investigaciones Científicas y Técnicas (CONICET), Universidad Nacional de Villa María (UNVM), Villa María X5220XAO, Argentina; mamontenegro@conicet.gov.ar (M.A.M.); noeliavanden@gmail.com (N.L.V.B.)
4 Instituto de Bionanotecnología del NOA (INBIONATEC), Consejo Nacional de Investigaciones Científicas y Técnicas CONICET, Universidad Nacional de Santiago del Estero (UNSE), Santiago del Estero G4206XCP, Argentina
* Correspondence: vipzanini@unse.edu.ar (V.I.P.Z.); nfferreyra@unc.edu.ar (N.F.F.); Tel.: +54-385-679-1166 (V.I.P.Z.); +54-351-535-3866 (N.F.F.)

Preparing of $Na^{\pm}$-Mt/GA-CHIT composite and GCE modification procedure

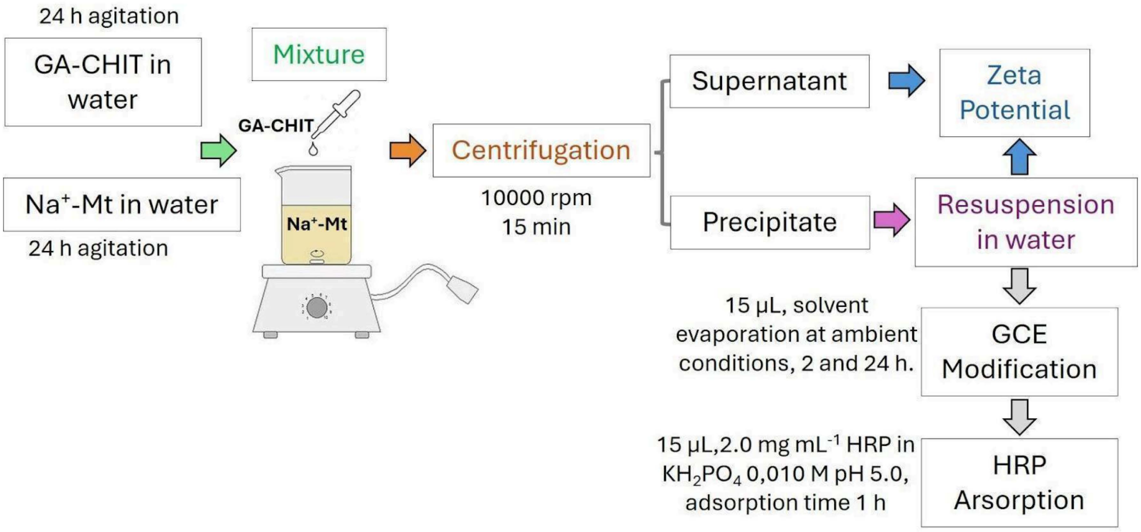

Scheme S1: Procedure used to obtain GCE/Na^+-Mt/GA-CHIT/HRP.

Se sitivity of the bioelectrodes as function of $Na^{\pm}$-Mt/GA-CHIT mass ratio

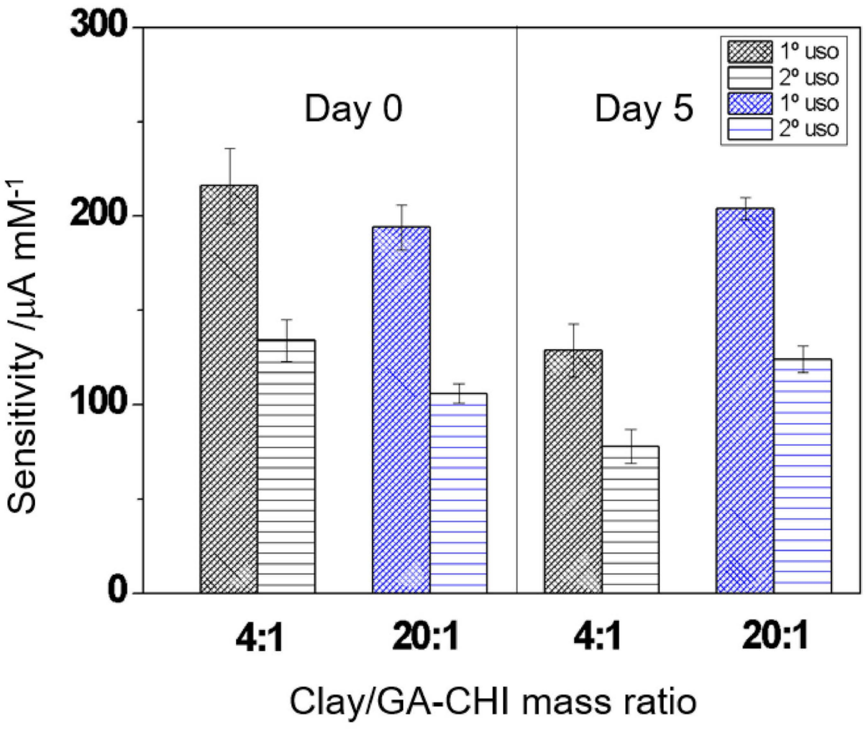

Figure S1 and Table S1: variation of the sensitivity of GCE/Na^+-MT/GA-CHIT/HRP as a function of the composite aging. A fresh solution of HRP was used for the

first experiment each day, while second use corresponds to the successive use with the same electrode (time between measures approximately 30 min).

Table S1

Na^+-Mt/GA-CHIt mass ratio	Sensitivity (µA/µM)* Fresh composite		Sensitivity (µA/µM)* Composite prepared 5 days before	
	First use	Second use	First use	Second use
4:1	(0.22 ± 0.02)	(0.13 ± 0.01)	(13±1)	(0.78±0.09)
20:1	(0.19 ± 0.01)	(0.106 ±0.005)	(0.204±0.006)	(0.124±0.007)

*Fresh solution 2.0 mg/mL of HRP was used.

<u>Immobilization of HRP at the modified GCE</u>

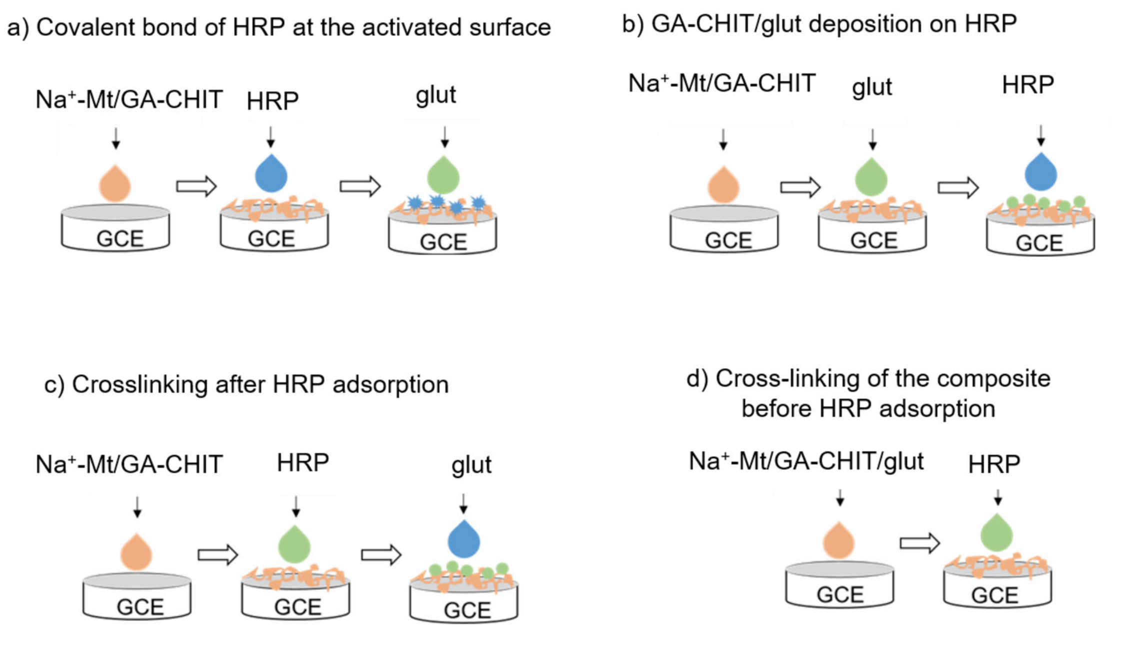

Scheme S2: Methodologies used for the immobilization of HRP with GLUT.

a) Activation of the amine groups exposed at the surface of GCE/Na^+-Mt/GA-CHIT for 30 min with a 0.125% V/V GLUT solution, rinsing with ultrapure water prior to the adsorption of HRP.

b) Mixture of a 0.125% V/V GLUT solution with 0.1 mg mL^{-1} of GA-CHIT followed by deposition on GCE/Na^+-Mt/GA-CHIT/HRP for 30 min and rinsing

with ultrapure water.

c) Physisorption of HRP at GCE/Na^{+}-Mt/GA-CHIT followed by cross-linking with a 0.125% V/V GLUT solution during 30 min and rinsing with ultrapure water.

d) Activation of the amino groups available at the Na^{+}-Mt/GA-CHIT composite by physical mixing with a 0.125% V/V (d1) or a 0.250%V/V (d2) of GLUT solution, followed by modification of the GCE and adsorption of HRP.

<u>Blank experiments</u>

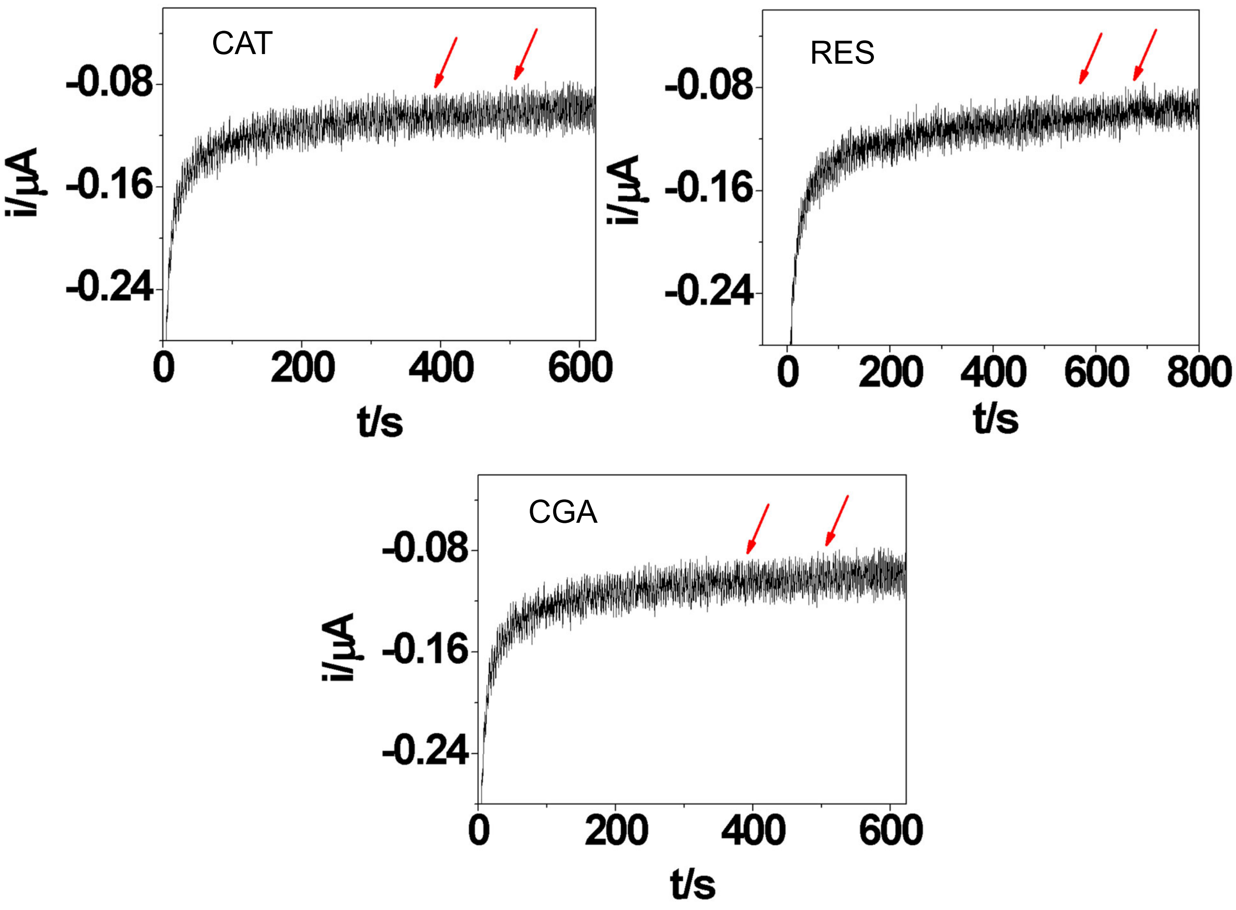

Figure S2: (A) Amperometric response towards CAT, RES and CGA at GCE/Mt/GA-Quit. Eap= -0.200 V; Supporting electrolyte: H_2O_2 600 μM in KH_2PO4 0.10 M pH 5.0. Current responses for 1, 2, 3 and 5 μM (a) and (b); and 10, 20, 30 and 50 μM (c) and (d) for the respective polyphenol.

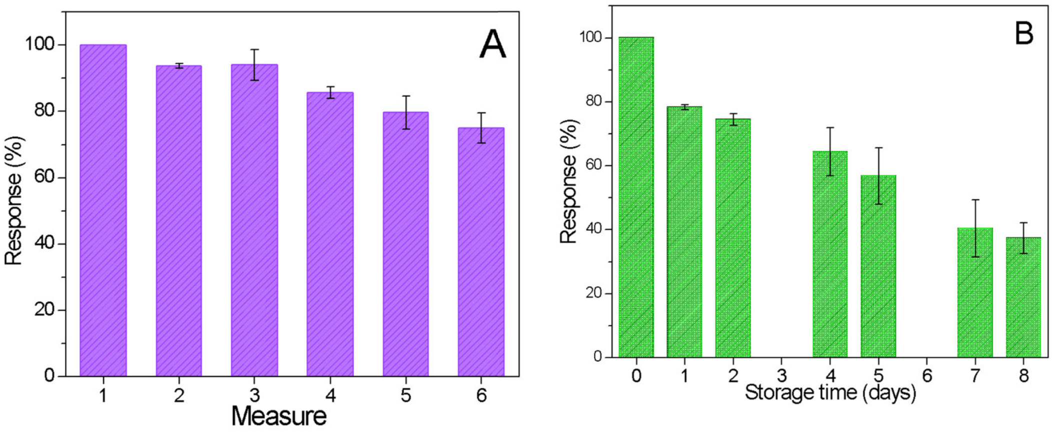

Figure S3: Variation of the biosensor sensitivity towards HQ for successive use (A) and over the time (B). E_{ap}= -0.200 V; Supporting electrolyte: H_2O_2 600 μM in KH_2PO4 0.10 M pH 5.0.

<u>Eady-Hoftee plots</u>

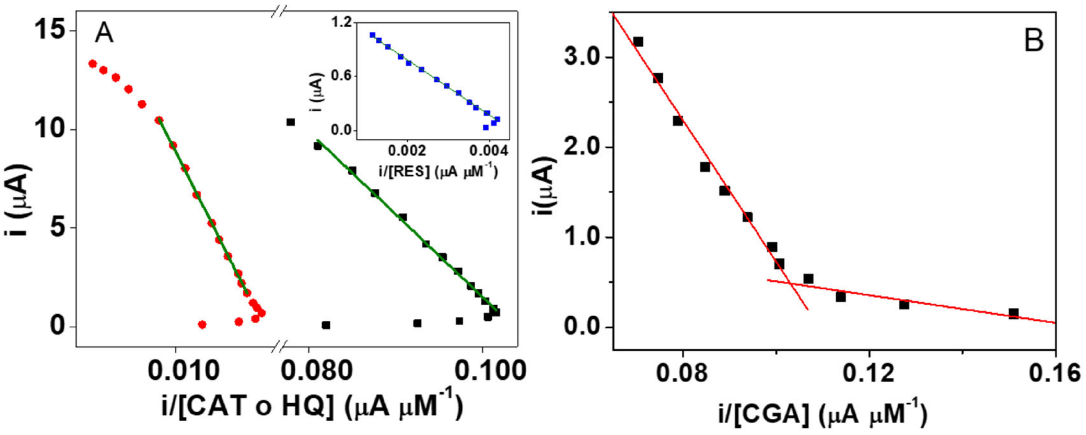

Figure S4: Eadie-Hofstee plot obtained at GCE/Na^{+}-Mt/GA-CHIT/glut/HRP for A) CAT (red), HQ (black), and RES (blue, inset) and B) CGA . Support electrolyte H_2O_2 600 μM in KH_2PO4 0.10 M pH 5.0. E_{ap}= -0.200 V.
